# West Nile Virus Encephalitis in a Barbary Macaque (*Macaca sylvanus*)

**DOI:** 10.3201/eid1004.030675

**Published:** 2004-04

**Authors:** Rolf-Arne Ølberg, Ian K. Barker, Graham J. Crawshaw, Mads F. Bertelsen, Michael A. Drebot, Maya Andonova

**Affiliations:** * Toronto Zoo, Ontario, Canada; † Ontario Veterinary College, Ontario, Canada; ‡ Health Canada, Manitoba, Canada

**Keywords:** Barbary macaque, *Macaca sylvanus*, encephalitis, West Nile virus, immunohistochemistry, *Flaviviridae*

## Abstract

An aged Barbary ape (*Macaca sylvanus*) at the Toronto Zoo became infected with naturally acquired West Nile virus (WNV) encephalitis that caused neurologic signs, which, associated with other medical problems, led to euthanasia. The diagnosis was based on immunohistochemical assay of brain lesions, reverse transcriptase–polymerase chain reaction, and virus isolation.

West Nile virus (WNV) is an arbovirus in the *Flaviviridae* family, which may cause inapparent infection, mild febrile illness, meningitis, encephalitis, and death in birds and mammals, including humans (*1*–*3*). Wild birds are the principal reservoirs of WNV, and mosquitoes, especially *Culex* species, are the primary vectors (*4*). WNV has caused several epidemics in the last 10 years (*3*). In 1999, WNV was detected in New York City (*5*). In Canada, WNV was first documented in August 2001 (*6*). We provide the first report of a naturally acquired infection with WNV in a nonhuman primate.

On August 17, 2002, symptoms of acute neurologic disease were observed in a 25-year-old male, 10.5 kg, Barbary macaque (*Macaca sylvanus*) at the Toronto Zoo. The animal behaved normally when observed by zookeepers the day before. It was housed with 10 other Barbary macaques in an outdoor exhibit with access to indoor housing. The animal exhibited clinical signs of neurologic involvement including ataxia, shaking, a drooping lower lip, excessive salivation, decreased responsiveness to surroundings, and nystagmus. The animal was anesthetized for further evaluation and a limited additional neurologic examination. Minor deviations were noted in hematology and serum biochemistry ranges. Because of severe chronic arthritis and the marked neurologic signs, the animal was euthanized and postmortem examination was performed.

The examination showed moderate gingivitis, generalized severe muscle atrophy, and severe bilateral femorotibial osteoarthritis. Brain and meninges were normal on gross examination. Tissues were collected from all major organs and frozen at minus 20°C. Additional samples from all major organs were fixed in 10% buffered formalin. The fixed brain was cut transversely from the rostral end to the spinal cord, each segment being 0.5 cm to 1.0 cm thick. Representative samples were taken from cortex, thalamus, hippocampus, midbrain, colliculi, pons, obex, medulla oblongata, and cerebellum. The tissue samples were subsequently trimmed and processed for routine histopathology. West Nile Virus immunohistochemistry was prepared by using rabbit polyclonal anti-WNV antiserum (Hana Weingartl, National Center for Foreign Animal Disease, Canadian Food Inspection Agency). Goat anti-rabbit immunoglobulin conjugated to a horseradish peroxidase (HRP)-labelled polymer (EnVision HRP, DAKO Cytomation, Inc., Missisauga, Ontario, Canada) was used as the secondary antibody. Nova Red (Vector Laboratories Canada Inc., Burlington, Ontario) was used as chromogen, and tissues were counterstained with Harris hematoxylin (Fisher Scientific, Toronto, Ontario). For negative controls, nonimmune rabbit serum was substituted for WNV antiserum.

Microscopically, a severe nonsuppurative meningoencephalitis was characterized by generalized gliosis, scattered glial nodules, and perivascular lymphoplasmacytic cuffing. The distribution and severity of lesions were bilaterally symmetrical. The brainstem, pons, colliculi, and cerebellum had the most extensive lesions, the anterior cortex and midbrain were moderately affected, and the posterior cortex mildly affected. Moderate mononuclear infiltrates and edema were present throughout the meninges, most severely over the cerebellum.

Immunohistochemistry for WNV was positive. WNV antigen was present in cerebellar Purkinje cells, neurons, and glial cells within or adjacent to sites of inflammation in the cerebellum, midbrain, and hypothalamus. A few individual glial cells in the cerebral cortex were sparsely stained. Viral antigen was not evident in liver, lymph node, lung, or kidney tissues. Other histopathologic findings included generalized moderate hepatocellular atrophy and focal to diffuse aggregates of lymphocytes, plasma cells, and macrophages, some of which had centers of caseous necrosis, in the renal interstitium.

WNV in brain tissue was also documented by the detection of viral genome by using real-time reverse transcriptase–polymerase chain reaction (RT-PCR) and by virus isolation. Extracted RNA was added to TaqMan PCR reaction (PE Applied Biosystems, Foster City, CA) mixtures containing primers and probes specific for the WNV (North American genotype) envelope gene and the 3′ nontranslated region (*7*). Viral RNA was amplified by using an ABI 7700 Sequence detector.

Diluted brain homogenates were used to infect monolayers of Vero cells at 80% confluence. Viral cytopathologic changes were observed 3 days post infection. WNV isolation was confirmed by immunofluorescence using chicken anti-WNV polyclonal sera and one-step RT-PCR of infected tissue culture supernatants.

The serum sample was positive for WNV on hemagglutination inhibition (HI) assays (*8*) with a low titre (1:40). The serum sample was also tested with some of the WNV enzyme linked immunosorbent assay (ELISA) developed for humans. The Centers for Disease Control and Prevention (CDC) immunoglobulin (Ig) M ELISA and the PanBio (Columbia, MD) IgM ELISA produced equivocal results and the PanBio IgG ELISA was negative.

Thirty-three primates in outdoor exhibits at the zoo were tested for WNV. Serum samples, taken in late December 2002 or spring 2003, were tested for antibodies to WNV by HI assay, and subsequently by plaque reduction virus neutralization (PRVN) if the hemagglutination inhibition test was positive (*8*). One of seven olive baboons (*Papio cynocephalus anubis*) had a titre on HI (1:320) and on PRVN (1:80). Two of 16 Japanese macaques (*Macaca fuscata*) had titres on HI (1:160 and 1:20), but only the first of these had a titre on PRVN test (1:40). None of the remaining 10 Barbary apes had serologic reactions.

Clinical signs in our study were similar to those observed in experimentally infected cynomolgus macaques (*Macaca* *fascicularis*) and rhesus macaques (*Macaca* *mulatta*) injected intracerebrally with the Egypt-101 strain of WNV, and in rhesus macaques injected intrathalamically using 10 different strains and mutants of WNV (*9*,*10*). Neurologic signs increased for several days and ptosis, paresis of the extremities and sphincters, adynamia, and marked hypothermia, were observed. If the animals did not die, they went into remission over the following 2 weeks.

The histologic lesions in our studies were similar in morphology findings and distribution to those described in experimentally infected macaques (*9*,*10*) and in severe cases in humans (*11*,*12*). A number of strains of WNV are capable of long-term persistence in nonhuman primates (*10*). In these animals the pathogenicity or neuroinvasiveness of the virus decreased with time. Some animals carried the virus for as long as 5 1/2 months, suggesting that primates might be carriers of WNV in foci of infection. These findings should be taken into account when using these animals as sources for cell cultures. A persistently infected nonhuman primate is unlikely to contribute to the WNV-mosquito cycle. Whether a persistently-infected non-human primate could be a source of infection for conspecific cage mates or people through bite wounds and scratches is speculative. Persistent infection with WNV has not been reported for other mammals.

The Barbary macaque in this case was probably immunocompromised to some degree, as it was aged and in poor physical condition; however, B-dependent splenic follicles were not atrophic. Hepatocellular and pancreatic atrophy, and poor physical condition, indicated reduced food intake. In humans, increasing age is considered a significant predisposing factor for more severe clinical disease in WNV infection (*13*).P ^P^

The prognosis for clinically affected nonhuman primates is difficult to predict based on one affected animal. However, a wide variance occurred in responses in experimentally infected macaques (*10*). The factors, except increased age, that predispose people to clinical disease are unknown (*13*). Resolution of the lesions is believed to be complete in human survivors of WNV meningoencephalitis, but for reasons poorly understood, permanent neurologic sequelae occur in some persons (*3*).

Since most cases in humans are mild or asymptomatic, such is probably the case in nonhuman primates, i.e., the clinically normal seropositives at the zoo. Infection may be prevalent but subclinical in nonhuman primates housed outdoors, or with access to outdoor holdings, in disease-endemic areas (*14*). Keeping the animals inside, especially infants and old adults, may be important when the virus is prevalent in surrounding mosquito populations or during periods of peak mosquito activity.

The Barbary ape was the first clinical case of WNV infection in any species recognized at the Toronto Zoo where WNV subsequently caused disease in a variety of avian species. WNV infection should be included as a differential diagnosis in all cases of muscle weakness and neurologic signs in nonhuman primates housed with outdoor access in WNV-endemic areas during the mosquito season. Virologic or serologic confirmation should be obtained in all suspected cases of WNV in nonhuman primates. Since the viremic stage is short, exposure is more reliably confirmed with paired serum samples demonstrating an increase in antibody to WNV (*14*), usually by HI, confirmed by plaque reduction virus neutralization. Neither of these tests are “species limited” in that they can be used in animals for which specific test reagents are not available (*3*,*15*). However, plaque reduction virus neutralization requires biosafety-level 3 facilities. Notably, IgM ELISA tests used in humans gave equivocal or negative results in this case. Laboratory findings and diagnostic techniques used in people are found in several articles (*3*,*15*).

**Figure 1:**
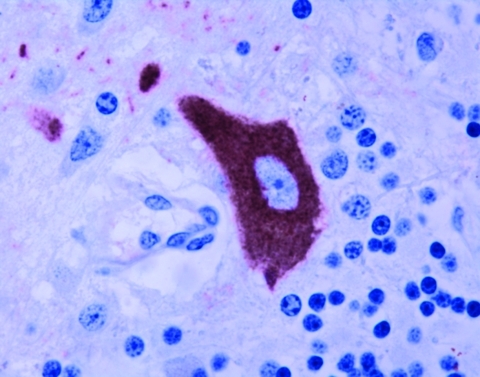
Staining of West Nile virus antigen in the cytoplasm of a Purkinje cell in the cerebellum. Immunohistochemistry. 40x.

**Figure 2:**
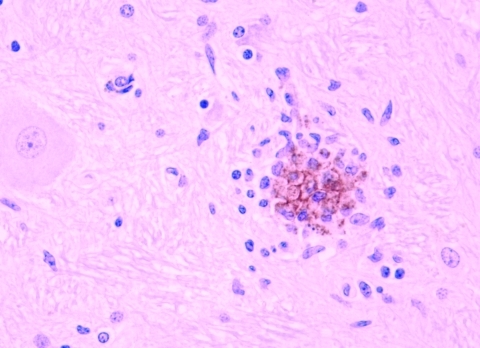
Staining of West Nile virus antigen in a glial nodule in the brainstem. Immunohistochemistry. 20x.
